# Emerging Therapies for Sickle Cell Disease: From Symptom Management to Curative Gene Therapy

**DOI:** 10.7759/cureus.95112

**Published:** 2025-10-21

**Authors:** Max S Duesberg, Gary Schiller

**Affiliations:** 1 Internal Medicine, Santa Clara Valley Medical Center, San Jose, USA; 2 Hematology and Oncology, University of California, Los Angeles, Los Angeles, USA

**Keywords:** crispr-cas9-mediated gene editing, gene therapy, hemoglobinopathies, lentiviral vectors, sickle cell disease

## Abstract

Sickle cell disease (SCD) is a hereditary hemoglobinopathy caused by a point mutation in the β-globin gene, leading to the production of hemoglobin S and resulting in chronic hemolytic anemia, vaso-occlusion, and progressive organ damage. Affecting millions globally, with the highest prevalence in sub-Saharan Africa and other low-resource settings, SCD remains a major public health challenge. Current therapies, including hydroxyurea, L-glutamine, crizanlizumab, and transfusions, primarily offer symptomatic relief but do not correct the underlying genetic defect. Hematopoietic stem cell transplantation remains the only established cure but is limited by donor availability and associated risks. Recent advances in gene therapy have transformed the therapeutic landscape of SCD, offering curative potential through techniques such as lentiviral vector-mediated gene addition and CRISPR/Cas9 gene editing. These approaches aim to restore normal hemoglobin production or reactivate fetal hemoglobin expression. While clinical trial outcomes are encouraging, with reduced vaso-occlusive crises and transfusion independence, major challenges remain, including high costs, need for myeloablative conditioning, and limited access in high-burden regions. This review explores the evolution of SCD treatment, evaluates the promise and limitations of emerging gene therapies, and highlights the urgent need for equitable access to these transformative technologies.

## Introduction and background

Overview of sickle cell disease 

Sickle cell disease (SCD) is a genetically inherited disorder characterized by a single point mutation in the β-globin gene, resulting in the substitution of valine for glutamic acid at the sixth position of the β-globin chain. This seemingly minor alteration leads to the production of hemoglobin S (HbS), which, under deoxygenated conditions, polymerizes and deforms red blood cells into a rigid, sickle shape. These abnormally shaped cells obstruct microvasculature, contributing to painful vaso-occlusive episodes, chronic hemolysis, and multiorgan dysfunction [1].

SCD affects approximately 100,000 individuals in the United States and millions globally, with the highest prevalence in sub-Saharan Africa, India, the Mediterranean, and the Middle East. Annual global births with SCD are estimated at around 300,000, making it a major contributor to morbidity and mortality in affected regions. While newborn screening programs facilitate early diagnosis in high-income countries, many individuals in low-resource settings remain undiagnosed until symptomatic [2-5].

Current standard therapies include hydroxyurea, which induces fetal hemoglobin (HbF) production to inhibit HbS polymerization, as well as L-glutamine and crizanlizumab, both of which mitigate the frequency and severity of vaso-occlusive crises. Transfusion therapy remains a cornerstone for acute and chronic complications but carries risks such as iron overload and alloimmunization. Voxelotor (Oxbryta) was previously used to increase hemoglobin levels but has since been withdrawn due to safety concerns pertaining to post-marketing data, which demonstrated an imbalance in vaso-occlusive crises and mortality amongst treated patients, raising significant safety concerns. This decision followed a reassessment of voxelotor’s benefit-risk profile, with the totality of clinical and post-marketing data indicating that the overall benefit no longer outweighed the risks in the approved SCD patient population. As a result, distribution and clinical studies of voxelotor have been discontinued pending further review and investigation as regulatory authorities formally reassess its benefits-risk profile. Bone marrow transplants offer a potential cure but are limited by donor availability and associated risks. More recently, gene therapy has emerged as a promising curative strategy by introducing functional hemoglobin genes or reactivating fetal hemoglobin production through genetic modification of the patient’s own stem cells, although challenges such as conditioning requirements and long-term safety remain [5-7].

Limitations of existing treatments

Current treatments, such as hydroxyurea and L-glutamine, primarily focus on palliation rather than curing SCD, as they reduce the frequency of pain crises, but do not address the underlying genetic defect. Pain management often requires opioids, which can lead to dependency issues and hyperalgesia [1,7-11].

While BMT is potentially curative, it is limited by the need for HLA-matched donors, which are available for only 14-20% of patients. In addition, there are risks of graft-versus-host disease, graft rejection, and transplantation-related mortality. These factors limit bone marrow transplants as a viable option for elderly patients with significant organ damage [9,12-15].

## Review

Mechanism of SCD

SCD arises from a single-nucleotide substitution in the HBB gene, wherein valine replaces glutamic acid at the sixth position of the β-globin chain. This molecular lesion leads to the production of HbS, a structurally abnormal hemoglobin variant with a unique biophysical property: its propensity to polymerize under deoxygenated conditions. The intracellular polymerization of deoxygenated HbS initiates a sequence of conformational changes that distort erythrocytes into rigid, sickle-shaped cells with impaired deformability and increased fragility. These biomechanical alterations set the stage for a complex disease process defined by microvascular occlusion, hemolysis, and progressive organ dysfunction [1,16-22,23-27].

The multifactorial pathophysiology of SCD centers on two dominant processes: vaso-occlusion and hemolytic anemia. Vaso-occlusion is precipitated by the entrapment of deformed, adhesive sickled erythrocytes within the microcirculation, impeding blood flow and generating ischemia-reperfusion injury. These interactions are mediated by upregulation of adhesion molecules on both the red blood cell membrane and vascular endothelium, fostering cellular aggregation and endothelial activation. Concurrently, ongoing hemolysis, both intravascular and extravascular, contributes to a chronic anemic state while liberating free hemoglobin and heme into the plasma. These byproducts act as potent pro-oxidants, promoting nitric oxide depletion, endothelial dysfunction, and inflammatory vasculopathy that drive long-term complications [23-27].

Compounding these core mechanisms are intrinsic defects in red cell membrane biology and oxidative homeostasis. Sickled erythrocytes exhibit externalization of phosphatidylserine, a procoagulant phospholipid, enhancing thrombin generation and accelerating splenic clearance by macrophages. Disruption of redox balance within these cells, marked by excessive reactive oxygen species and diminished antioxidant defenses, destabilizes the membrane and magnifies inflammatory signaling. Hemorheological changes within the microcirculation further amplify vascular injury: the rigid, adhesive sickled cells alter laminar flow dynamics and increase shear stress on the endothelium, creating a microenvironment of localized hypoxia. Thus, the cyclical nature of oxygen tension within the circulation perpetuates recurrent episodes of sickling and unsickling, leading to cumulative membrane injury, cellular dehydration, and premature erythrocyte destruction [23-27].

Gene therapy approaches for SCD

Therapeutic strategies employing gene therapy for SCD have converged on two principal modalities: gene addition and gene editing. Both paradigms involve the autologous collection of hematopoietic stem and progenitor cells (HSPCs), ex vivo genetic modification, and subsequent reinfusion following myeloablative conditioning, most commonly with busulfan. These interventions aim to correct the pathogenic consequences of the β-globin gene mutation either by introducing a functional transgene or by altering endogenous gene expression to favor antisickling hemoglobin production [16-22].

Gene Addition Therapy

Gene addition therapy leverages integrating viral vectors, typically lentiviruses, to insert functional copies of the HBB gene into the genome of autologous HSPCs. The resultant erythroid progeny express engineered hemoglobin variants designed to recapitulate or mimic normal hemoglobin function, thereby counteracting the polymerization tendency of HbS. The most clinically mature example of this approach is lovotibeglogene autotemcel (lovo-cel; formerly BB305 or bb1111), developed by Bluebird Bio [17].

In the HGB-206 phase 1-2 clinical trial, 35 patients with severe SCD received a single infusion of lovo-cel following myeloablation with busulfan. The study demonstrated sustained and clinically significant hematologic reconstitution, with a median follow-up of 17.3 months. Hemoglobin levels increased from a baseline median of 8.5 g/dL to ≥11 g/dL starting approximately six months post-infusion and were maintained through three years of follow-up. Importantly, a significant proportion of total hemoglobin was composed of HbAT87Q, a synthetic anti-sickling variant, which accounted for ≥40% of total hemoglobin in most recipients, a level associated with amelioration of sickling pathophysiology. Among evaluable patients, those with a documented history of frequent VOCs prior to treatment achieved complete resolution of these events, highlighting the therapy’s impact on one of the most severe and disabling manifestations of the disease [17].

Additional hematologic improvements included reductions in hemolytic biomarkers, increased hemoglobin stability, and decreased healthcare utilization due to fewer hospitalizations. The safety profile of lovo-cel was acceptable, with no evidence of replication-competent lentivirus, insertional oncogenesis, or clonal dominance. Adverse effects were primarily attributed to the conditioning regimen, with no gene therapy-specific toxicities emerging. These findings position gene addition as a viable and potentially curative approach for a subset of patients with severe SCD, pending broader validation in larger and longer-term studies [17].

Gene editing techniques

CRISPR/Cas9 for HBB Mutation Correction

OTQ923 (trial: phase I/II; regulatory status: investigational): One of the most precise and promising gene editing strategies in development for SCD is direct correction of the causative HBB mutation using CRISPR/Cas9. This approach forms the basis of OTQ923, an investigational therapy developed by Vertex Pharmaceuticals and CRISPR Therapeutics. The strategy involves isolating autologous HSPCs and introducing a targeted double-strand break at the site of the pathogenic point mutation in the HBB gene. Through homology-directed repair (HDR), and with the aid of an exogenous DNA repair template, the sickle mutation is precisely replaced with the wild-type nucleotide sequence. The corrected stem cells are then reinfused into the patient following myeloablative conditioning, enabling reconstitution of the hematopoietic system with cells capable of producing structurally and functionally normal adult hemoglobin [18].

This targeted correction strategy offers a potential one-time cure by restoring normal adult hemoglobin expression. In a phase 1-2 clinical study, three adults with severe SCD received autologous OTQ923 following busulfan-based conditioning. Over a follow-up period of six to 18 months, all participants exhibited stable hematologic recovery and sustained induction of HbF, a beneficial secondary effect that further reduced hemoglobin polymerization and red blood cell sickling. Clinically, each individual experienced meaningful improvement in disease severity, although the small sample size and early stage of evaluation limit the ability to draw conclusions regarding long-term efficacy and safety [18].

Nevertheless, these early findings provide proof of principle for the feasibility of precise gene correction in SCD. OTQ923 represents an important step forward in gene editing therapeutics and demonstrates the potential for curative intervention through direct molecular repair of the underlying genetic defect [18].

Limitations of the OTQ923 gene editing trial

The OTQ923 phase 1-2 trial enrolled a small, selectively screened cohort of adults with severe SCD who were eligible for myeloablative conditioning and autologous stem cell collection. Patients with significant comorbidities, advanced organ dysfunction, chronic opioid use, or persistent pain syndromes were excluded, limiting applicability to the broader SCD population [18].

Trial endpoints focused on safety, engraftment, and HbF induction, with clinical outcomes such as vaso-occlusive crisis (VOC) frequency reported descriptively. VOC definitions were not standardized, and the small sample size complicates cross-trial comparisons, particularly with studies using different efficacy endpoints [18].

Follow-up data were limited to just three participants, with a maximum duration of 18 months. This short observation period precludes conclusions about long-term safety, durability of response, and risks of late-onset complications, critical considerations for genome-editing therapies in SCD [18].

Base editing

Base editing represents a novel gene correction strategy for SCD that enables precise, single-nucleotide alterations without the need for double-stranded DNA breaks or exogenous donor templates. This approach utilizes a catalytically impaired Cas9 enzyme fused to a nucleotide deaminase to induce direct base conversions at the DNA level. In the case of SCD, adenine base editors (ABEs) are designed to convert the pathogenic adenine (A) within the Glu6Val mutation (GAG → GTG) in the HBB gene to a guanine (G), thereby creating a non-pathogenic variant, such as the Makassar variant (GCG), that does not induce sickling. By avoiding double-strand breaks, base editing minimizes risks associated with traditional nuclease-based editing platforms, including chromosomal rearrangements, large deletions, and activation of the DNA damage response. This potentially translates into a superior safety profile while maintaining high editing precision and efficiency. Early preclinical studies have demonstrated successful correction of the sickle mutation, restoration of normal hemoglobin production, and reversal of red blood cell sickling phenotypes in edited hematopoietic stem and HSPCs. Although still in the preclinical phase, base editing holds considerable promise as a next-generation therapeutic strategy for SCD, offering the potential for curative intervention through highly specific and minimally invasive genomic modification [28-37].

Future considerations

CRISPR/Cas9-based gene editing for correction of the HBB mutation in SCD faces several challenges related to feasibility, efficiency, efficacy, and long-term safety. Efficient delivery of the CRISPR/Cas9 system into HSPCs remains a critical hurdle, with various delivery platforms exhibiting variable success rates and potential toxicity. While some studies have reported allelic correction rates as high as 60%, editing efficiency depends heavily on the delivery method and the specific CRISPR/Cas9 construct used. Efficacy is evidenced by reductions in red blood cell sickling and increased production of normal hemoglobin after editing. However, sustained long-term efficacy requires further validation, particularly regarding the durability of edited cells and concerns over potential myeloid lineage bias during engraftment. In addition, safety considerations include the risk of off-target mutations and chromosomal rearrangements, which could result in genotoxic effects. Activation of DNA damage responses and inflammatory pathways in edited cells also raises potential safety concerns. Collectively, these challenges underscore the need for continued optimization and rigorous evaluation of CRISPR/Cas9 gene editing approaches to ensure their safe and effective application in SCD therapy [1,10-11,13,38-48].

HbF Reactivation

BCL11A Gene Suppression: BCH-BB694. Trial: Early Phase I. Regulatory Status: Investigational.

Gene therapy approaches aimed at reactivating HbF focus on suppressing BCL11A, a key transcriptional repressor of HbF expression. By editing regulatory regions of the BCL11A gene, it is possible to induce sustained HbF production, which can alleviate the clinical manifestations of SCD. Editas Medicine’s investigational therapy, BCH-BB694, employs this strategy and has demonstrated promising results in early-phase clinical trials. In one study, six patients with severe SCD received BCH-BB694 following myeloablative conditioning, resulting in robust and stable induction of HbF alongside significant clinical improvement [49].

However, the trial included a small, carefully selected patient population, excluding individuals with significant comorbidities, advanced organ dysfunction, or chronic opioid dependence, which limits the broader applicability of the findings. The study’s primary endpoints focused on safety, stem cell engraftment, and HbF induction, while clinical outcomes such as VOCs were reported descriptively without standardized definitions. The small sample size and lack of uniform VOC criteria constrain cross-trial comparisons and broader interpretation of efficacy [49].

Follow-up data currently extend to a median of 18 months (range seven to 29 months) among the six participants, a timeframe insufficient to fully assess long-term safety, durability of response, and potential late adverse effects. These factors remain critical to establishing the long-term clinical utility of gene therapies targeting BCL11A for SCD [49].

Alternative Gene Therapy Approaches

RNA-based therapies: RNA-based gene therapy for SCD represents a non-integrative strategy aimed at transiently manipulating the genetic program of HSPCs. Synthetic RNA molecules, including messenger mRNA encoding gene-editing enzymes or guide RNAs, are introduced into HSPCs to direct targeted modification of the pathogenic HBB allele. This platform enables precise, temporary correction at either the mRNA or DNA level without permanent genomic integration. Although inherently transient, RNA-based strategies may minimize risks associated with insertional mutagenesis and offer a flexible, repeatable therapeutic modality in the evolving landscape of gene editing technologies [28,32,34].

Injection of a viral vector: In vivo gene therapy utilizes systemic administration of viral vectors, most commonly lentiviruses, to deliver therapeutic transgenes directly into HSPCs within the patient’s bone marrow niche. These vectors typically carry a modified β-globin transgene capable of encoding antisickling hemoglobin variants that interfere with HbS polymerization. By avoiding ex vivo manipulation and transplantation, this method simplifies the therapeutic workflow and has the potential to broaden access. Clinical trials employing this technique have demonstrated durable expression of therapeutic hemoglobin and significant clinical benefits, including resolution of vaso-occlusive episodes, improvement in hemolytic parameters, and enhanced hematologic function. Importantly, this approach circumvents the immunologic complications associated with allogeneic transplantation [28,50-56].

Current clinical trials and milestones

Gene therapy for SCD has reached a pivotal stage, with clinical trials demonstrating durable efficacy. Two leading approaches, i.e., lovotibeglogene autotemcel (lovo-cel) and exagamglogene autotemcel (exa-cel), have shown sustained increases in hemoglobin, resolution of severe vaso-occlusive events, and transfusion independence in most patients. These results reflect the potential for gene therapy to alter the disease course fundamentally.

LentiGlobin (Bluebird Bio)

Lovotibeglogene autotemcel (lovo-cel; bb1111) (trial: phase I/II HGB-206; regulatory status: investigational in the U.S., approved in the EU for β-thalassemia only (not SCD)): Lovo-cel utilizes a lentiviral vector to introduce a modified β-globin gene (HbA^T87Q) into autologous hematopoietic stem cells, enabling production of antisickling hemoglobin. In the HGB-206 study, 28 of 32 evaluable patients experienced complete resolution of severe VOEs, with sustained HbAT87Q expression and reduced hemolysis markers [44,46-47,57-58].

Limitations of the LentiGlobin Trial

The lovo-cel (LentiGlobin) trial is subject to several limitations that affect the generalizability and long-term interpretability of its findings. The study cohort was selectively enriched for younger, relatively healthy individuals, excluding patients with significant comorbidities, chronic opioid use, or poor performance status, criteria that limit relevance to the broader sickle cell population. In addition, variability in the definition of severe VOEs, including inconsistent inclusion of events such as hospitalization, acute chest syndrome, or priapism, introduces trial heterogeneity that complicates direct comparisons with other gene therapy studies. While follow-up of up to 87 months has been reported, the median duration remains approximately 3.5 years. Consequently, long-term risks such as insertional oncogenesis, clonal expansion, and delayed toxicities remain incompletely characterized and require ongoing surveillance [17].

Exa-Cel (Vertex and CRISPR Therapeutics)

Exagamglogene autotemcel (exa-cel) (trial: phase I/II; regulatory status: approved by the FDA and UK MHRA for SCD and transfusion-dependent β-thalassemia (2023-2024)): Exa-cel employs CRISPR-Cas9 to disrupt the erythroid enhancer of BCL11A, reactivating HBF production by targeting the HBG1 and HBG2 promoters in autologous hematopoietic stem cells. In early-phase trials, this gene-editing strategy has yielded robust and sustained HbF induction. Among 30 evaluable patients with SCD, 29 remained free from severe vaso-occlusive crises for at least 12 months post-treatment, with concurrent improvements in hemoglobin levels and quality of life. Parallel studies, including OTQ923, have also demonstrated successful HbF reactivation and clinical benefit in early cohorts [18, 59-61].

Limitations of the Exa-Cel Trial

The exa-cel clinical program has important limitations that affect generalizability and long-term risk assessment. Trials enrolled patients aged 12-35 with severe SCD or transfusion-dependent β-thalassemia (TDT), excluding individuals with advanced organ dysfunction, frequent chronic pain hospitalizations, or matched related donors. These restrictive criteria limit applicability to patients with more complex or refractory disease. In addition, trial heterogeneity complicates interpretation: primary endpoints varied between disease groups, freedom from severe VOCs in SCD and transfusion independence in TDT, while VOC definitions and adjudication methods were not standardized across studies. Although some participants have been followed for up to four years, the median follow-up remains under two years. Long-term risks such as clonal hematopoiesis, oncogenic transformation, or late toxicities remain theoretical but unresolved, pending the completion of ongoing 15-year observational studies [18,59-62].

Time to Hematopoietic Recovery and Kinetics of Erythrocyte Engraftment

The evaluation of hematopoietic recovery and erythrocyte engraftment kinetics is pivotal for assessing the efficacy and safety of gene therapy interventions in SCD. These parameters reflect the functional reconstitution of the bone marrow compartment and the subsequent restoration of erythropoiesis following myeloablative conditioning and infusion of genetically modified HSCs.

In the LentiGlobin clinical trial, hematopoietic reconstitution was characterized by a median neutrophil engraftment time of 18 days (range 14-25 days), indicating rapid myeloid lineage recovery. Platelet engraftment followed at a median of 35 days (range 20-50 days), consistent with effective megakaryocytic restoration. This timely engraftment facilitated sustained production of antisickling hemoglobin variant HbAT87Q, which correlated with reductions in hemolytic biomarkers, confirming functional erythrocyte recovery and disease amelioration [18].

Similarly, CRISPR-Cas9-based gene editing strategies, including those employed in OTQ923 and exagamglogene autotemcel (exa-cel) trials, demonstrated comparable hematopoietic kinetics. The OTQ923 trial reported genetically modified HSC engraftment within 18 to 26 days, without evidence of lineage skewing, preserving normal differentiation into myeloid and B-cell compartments. The exa-cel trial documented a median neutrophil engraftment time of 27 days (range 15-40 days) and platelet recovery by a median of 35 days (range 23-126 days), underscoring robust multilineage hematopoietic recovery [47,59].

Collectively, these findings establish that both lentiviral vector-mediated gene addition and CRISPR-Cas9 gene editing achieve efficient and timely hematopoietic recovery and erythrocyte engraftment in SCD patients. This engraftment kinetics underpins durable therapeutic hemoglobin expression and represents a critical determinant of long-term clinical benefit in severe SCD.

Comparison of Trial Designs

Trial designs for both modalities (as described and contrasted in Table 1) involve autologous HSC collection, ex vivo modification, myeloablative conditioning, and reinfusion, but inclusion criteria, endpoints, and follow-up durations vary, with LentiGlobin trials often requiring a history of frequent vaso-occlusive events and CRISPR-Cas9 studies focusing on HbF induction and clinical event reduction [17,18]. Implementation challenges for both include the need for specialized manufacturing infrastructure, high cost, myeloablative conditioning toxicity, and psychosocial barriers to access and informed consent, particularly in historically marginalized SCD populations [17,18]. Both approaches are now FDA-approved, but real-world access, long-term monitoring, and health equity remain significant hurdles [17,18].

**Table 1:** Summary of all current gene therapy trials This table summarizes current gene therapy approaches for sickle cell disease (SCD), including gene addition, gene editing, fetal hemoglobin reactivation, and emerging in vivo strategies. Each therapy is defined by its mechanism of action, developmental stage, and clinical outcomes to date. LentiGlobin (bb1111; lovo-cel) and exagamglogene autotemcel (exa-cel) are the most advanced, demonstrating significant reductions in vaso-occlusive crises and transfusion needs. Early-phase trials and preclinical approaches, including base editing, prime editing, and RNA-based therapies, highlight the ongoing innovation aimed at delivering safe, durable, and accessible cures for SCD.

Therapy / trial name	Type	Mechanism	Stage	Clinical outcomes
LentiGlobin (bb1111; lovo-cel)	Gene addition	Lentiviral vector delivers anti-sickling β-globin gene (Hemoglobin A T87Q) into hematopoietic stem cells	Phase I/II (HGB-206)	Increase in hemoglobin ≥11 g/dL, decrease in hemolysis, 28/32 vaso-occlusive crisis resolution (evaluable patients), durable hemoglobin A T87Q expression [17]
Exagamglogene Autotemcel (exa-cel)	Gene editing	CRISPR-Cas9 disrupts B-cell lymphoma/leukemia 11A (BCL11A) erythroid enhancer to induce fetal hemoglobin	Phase I/II	29/30 patients vaso-occlusive crisis-free ≥12 months, increased hemoglobin, improved quality of life; approved in the United States/United Kingdom [61]
OTQ923	Gene editing	CRISPR/Cas9 corrects β-globin mutation via homology-directed repair in hematopoietic stem and progenitor cells	Phase I/II	Three patients with increased fetal hemoglobin and clinical improvement; 6–18 months follow-up; investigational [18]
BCH-BB694	Gene editing	Targets B-cell lymphoma/leukemia 11A regulatory regions to reactivate fetal hemoglobin	Early phase I	Six patients: robust fetal hemoglobin induction, decreased disease burden; investigational [49]
Base Editing (Makassar variant)	Gene editing	Converts pathogenic adenine→guanine in the β-globin gene to a non-pathogenic variant without double-strand breaks	Preclinical	High editing precision, reduced off-target effects, promising in murine/human hematopoietic stem and progenitor cells [31]
Prime Editing	Gene editing	Directs precise correction of the βS allele in vivo	Preclinical	Efficient correction in mouse models, increased adult hemoglobin (hemoglobin A), decreased sickling [34]
RNA-Based Therapies	Transient genetic modulation	Delivers messenger RNA or guide RNAs to correct the hemoglobin subunit beta gene (HBB) mutation or regulate expression	Preclinical	Under investigation; transient effects, avoids DNA breaks [28,32,34]
Stem Cell + Gene Modification	Combined cell/gene therapy	Genetically corrects hematopoietic stem cells and reinfuses autologously	Preclinical / pilot clinical	Aims for durable adult hemoglobin or fetal hemoglobin production; scalable curative intent [16]
In Vivo Viral Vector Injection	In vivo gene addition	Direct intravenous administration of vectors encoding β-globin variants	Early clinical / preclinical	Proof-of-concept in animal models; avoids ex vivo steps [28,50-56]

Challenges and barriers to implementation

Despite the transformative potential of gene therapy for SCD, several translational and logistical barriers limit its scalability and integration into routine clinical care. The complexity of delivering safe, effective, and timely autologous gene-modified cell products to patients with underlying hematologic fragility presents distinct scientific and clinical challenges.

Technical Challenges

Precision and efficiency of gene editing technologies: The therapeutic success of genome engineering approaches, such as CRISPR/Cas9, TALENs, and zinc-finger nucleases (ZFNs), relies on efficient and precise genetic modification of autologous HSPCs. While proof-of-concept studies have validated their capacity to correct the β-globin defect or derepress HbF, editing efficiency in primary HSCs remains variable. The rarity of long-term repopulating stem cells within the HSPC pool necessitates high fidelity and durable editing within a limited target population, a constraint that underscores the need for optimized delivery systems and ex vivo culture conditions to preserve stemness [63-65].

Off-target effects and genotoxicity: Unintended cleavage at non-target genomic loci poses a significant safety concern, with the potential for insertional mutagenesis, chromosomal rearrangements, or oncogene activation. These genotoxic risks are especially critical in autologous settings where edited cells contribute long-term hematopoiesis. Ongoing efforts aim to refine nuclease specificity through high-fidelity enzyme variants, improved guide RNA design, and non-viral delivery systems. Comprehensive preclinical and clinical monitoring strategies are essential to detect clonal dominance or early signs of malignant transformation [47,64].

Delayed hematopoietic recovery: The timeline for hematopoietic reconstitution following myeloablative conditioning and gene-modified cell infusion remains a critical determinant of early post-treatment morbidity. Prolonged neutropenia and thrombocytopenia expose patients to infection, bleeding, and hospitalization. These risks are exacerbated by the intensity of conditioning regimens such as busulfan, which, while necessary for engraftment, can cause severe mucositis, organ toxicity, and marrow aplasia. Strategies to expedite hematopoietic recovery include improved transduction efficiency, supportive care protocols, and reduced-intensity conditioning regimens under active investigation [66-67].

Risks of pre-treatment exchange transfusion: Exchange transfusion is frequently employed preconditioning to decrease circulating sickle hemoglobin and reduce the risk of peritransplant VOEs. However, this intervention introduces its own set of complications, including alloimmunization, iron overload, and delayed hemolytic transfusion reactions. The cumulative burden of transfusion exposure also strains resource-limited settings, adding logistical and financial challenges to gene therapy administration [17, 66].

Time to receipt of the transduced product: The production of autologous, gene-modified HSPC products is an intricate, multi-step process encompassing apheresis collection, ex vivo gene editing, cell expansion, quality control testing, and cryopreservation. This manufacturing timeline often spans several weeks, during which the patient must remain clinically stable. Delays in product release or failures in meeting potency or sterility criteria may necessitate re-collection or treatment postponement, thereby compounding clinical risk and emotional burden for patients and families [66,67].

Need for autologous products and procurement issues: Mobilization of functional HSPCs in patients with SCD presents unique biological constraints. G-CSF, the standard mobilization agent in other hematologic indications, is contraindicated due to its association with life-threatening vaso-occlusive complications in SCD. As a result, plerixafor, a CXCR4 antagonist, is used as a single agent for mobilization. However, interpatient variability in CD34+ cell yield often necessitates multiple collection attempts, increasing the risk of catheter-related complications such as bloodstream infections and thrombosis. The underlying bone marrow damage in chronically transfused patients further compounds the difficulty of procuring adequate autologous grafts [17,66].

Collectively, these challenges highlight the multifactorial nature of barriers to widespread implementation of gene therapy in SCD. Addressing them will require continuous refinement of gene editing platforms, conditioning strategies, manufacturing scalability, and patient selection protocols. Bridging the gap between clinical trial efficacy and real-world feasibility is essential for democratizing access to curative therapies.

Regulatory and Ethical Considerations

Ethical concerns: germline versus somatic gene editing: Gene therapy for SCD currently targets somatic cells, specifically autologous HSPCs, thereby confining genetic alterations to the treated individual. In contrast, germline editing, which introduces heritable genetic modifications, remains ethically contentious and is not employed in therapeutic contexts. The distinction carries profound ethical implications. Germline editing raises unresolved questions about intergenerational consent, unforeseen long-term consequences, and the potential for non-therapeutic applications or enhancement. Even in the context of somatic gene editing, ethical imperatives remain central. These include ensuring robust informed consent, particularly for pediatric or socioeconomically vulnerable populations, balancing risks and benefits in the absence of long-term safety data, and safeguarding against coercion or therapeutic misconception in clinical trial recruitment [68-71].

Regulatory pathways for gene therapy approval, differences across regions: The approval and oversight of gene therapies are governed by complex, region-specific regulatory structures that influence both the pace of clinical translation and the geography of access. In the United States, the Food and Drug Administration (FDA) has established expedited pathways for regenerative medicines, culminating in the recent approval of exagamglogene autotemcel (exa-cel) for SCD. Similarly, the UK Medicines and Healthcare products Regulatory Agency (MHRA) has advanced gene therapy approvals through its innovation-friendly frameworks. However, regulatory infrastructure in many low- and middle-income countries remains underdeveloped or misaligned with evolving genomic technologies, contributing to delays in approval, restricted access, and widening global disparities in availability of curative therapies [68-71].

In this context, international regulatory harmonization, capacity-building initiatives, and technology transfer agreements are essential to ensure that the benefits of gene therapy extend beyond high-resource settings. Ethical and regulatory vigilance must evolve in tandem with technological advancement to protect patient autonomy, uphold safety standards, and promote just distribution of transformative medical innovations.

Accessibility and Cost

High cost of gene therapies: The cost of gene therapies for SCD is prohibitively high, often exceeding $1.5 million per treatment. These costs limit access for patients in both high-income and especially low- and middle-income countries, where the majority of the global SCD burden exists. Even in high-resource settings, the financial impact on public payers such as Medicaid is substantial, with short-term budget impacts threatening the sustainability of widespread adoption and raising concerns about equitable access for marginalized populations [72-75].

The sustainability of these therapies is further challenged by the need for complex infrastructure, highly specialized personnel, and long-term follow-up, which are not universally available. Equity concerns are heightened by the risk that only a small subset of patients-those with access to advanced healthcare systems and robust insurance-will benefit, while others are left behind [72-77].

Efforts to reduce costs and improve access include technological innovations such as the development of in vivo gene therapy platforms, which aim to simplify manufacturing and delivery, potentially lowering costs and expanding reach. Policy interventions under discussion include alternative payment models (e.g., annuity or outcomes-based payments), public-private partnerships, and harmonized regulatory frameworks to facilitate approval and distribution in resource-limited settings. Multidisciplinary task forces have proposed academic licensing reforms, manufacturing innovation, and diversified funding sources as strategies to reduce per-patient costs and enable broader patient treatment. These combined approaches are critical to address the current limitations in accessibility, sustainability, and equity of gene therapies for sickle cell disease [72-77].

Access to therapy in low-income regions with high SCD prevalence: Equity and access to gene therapies for SCD are profoundly challenged by the disproportionate burden of SCD in sub-Saharan Africa and other low- and middle-income countries (LMICs), where over 75% of affected individuals reside and where the majority of SCD-related childhood mortality occurs. The high cost of gene therapies, often exceeding $1.5 million per treatment, renders them inaccessible in these settings and exacerbates global health inequities. Beyond cost, significant barriers include limited healthcare infrastructure, a shortage of qualified healthcare workers, lack of regulatory and ethical oversight, and inadequate access to even basic disease-modifying therapies such as hydroxyurea [2,78-80].

Current ex vivo gene therapy platforms require advanced laboratory and clinical resources that are largely unavailable in LMICs, making widespread implementation unfeasible. The absence of harmonized regulatory frameworks and the lack of local manufacturing capacity further impede access. Ethical concerns are heightened in these regions, where informed consent processes may be compromised by low health literacy, and where the risk-benefit ratio of novel therapies is difficult to assess in the context of limited standard care [2,78-82].

Efforts to address these disparities must include collaborative, multi-stakeholder approaches to build infrastructure, develop context-appropriate regulatory and ethical frameworks, and ensure community engagement. There is consensus in the medical literature that while gene therapy holds transformative potential, immediate priorities should include scaling up access to proven, affordable interventions, such as hydroxyurea and integrating SCD care into primary health systems, while simultaneously investing in the development of in vivo gene therapy platforms that could be more accessible and cost-effective for high-burden, resource-limited settings [81-82].

As the field addresses these barriers, ongoing innovation is shaping the future landscape of SCD treatment. The next section highlights emerging technologies and strategies that could enhance the accessibility and effectiveness of gene therapies.

Future directions and emerging technologies 

Enhancements in Gene Editing Technology

Recent advancements in gene editing technologies, including base editing and prime editing, have markedly improved the precision and safety profile of genetic interventions for SCD. Base editing technologies, which enable the conversion of specific DNA bases without introducing double-strand breaks, have shown promise in directly correcting the pathogenic HBB mutation responsible for SCD. In murine models and ex vivo human hematopoietic stem and progenitor cells, adenine base editors have achieved high rates of conversion of the sickle allele to non-pathogenic variants, resulting in significant reductions in hypoxia-induced sickling and restoration of near-normal hematological parameters [1,8,32,43].

Prime editing, a more recent innovation, allows for precise correction of the SCD mutation in hematopoietic stem cells in vivo. In mouse models, prime editing has achieved efficient and durable correction of the βS allele, with substantial replacement of sickle hemoglobin by adult hemoglobin and mitigation of SCD phenotypes, all with minimal off-target activity.

Collectively, these gene editing strategies represent a paradigm shift in the treatment of SCD, offering the potential for one-time, curative interventions that address the underlying genetic defect while minimizing genotoxicity and off-target risks [22,32,43,47].

Potential for Combined Therapies

The evolving landscape of SCD therapeutics increasingly supports a multidimensional approach, wherein gene therapy is combined with adjunctive pharmacologic or molecular agents to optimize clinical outcomes while mitigating associated risks. Such strategies aim to exploit mechanistic complementarity between interventions, reduce the required intensity of gene modification, and enhance the therapeutic index.

Combinatorial Genetic Engineering Approaches

Recent investigations have explored the integration of lentiviral vector-mediated gene addition with CRISPR/Cas9-based gene editing to achieve enhanced expression of antisickling hemoglobins at lower vector copy numbers. This dual-modality platform capitalizes on the robust transgene delivery of lentiviral systems while leveraging CRISPR-mediated disruption of endogenous repressors, such as BCL11A, to induce HbF synthesis. Preclinical data demonstrate that this combinatorial strategy results in synergistic augmentation of therapeutic hemoglobin levels, phenotypic correction of erythrocyte sickling, and attenuation of hemolysis, all at reduced genomic integration burden, thus lowering genotoxicity risks and improving long-term safety profiles [52].

Adjunctive Pharmacologic Agents

In parallel, adjunctive use of pharmacologic therapies continues to offer supportive or synergistic benefit alongside gene-based interventions. Agents such as hydroxyurea, which induces HbF expression, and L-glutamine, which reduces oxidative stress, may serve to temporize disease progression during the preparative or recovery phases of gene therapy. Similarly, pyruvate kinase activators and anti-adhesion therapies like crizanlizumab target downstream pathophysiologic pathways-improving red cell metabolism and reducing vaso-occlusive episodes, respectively. These agents may offer an additive or bridging benefit, particularly in patients awaiting definitive genomic correction or in those with suboptimal gene modification outcomes [52].

The therapeutic potential of integrated approaches, genetic and pharmacologic, offers a promising avenue to tailor treatment to individual patient profiles, reduce reliance on high-intensity conditioning, and ultimately extend the reach of curative strategies to broader populations. Future trials evaluating the pharmacodynamic interplay and long-term impact of such combinations will be essential to validate these paradigms and refine risk-benefit calculations for precision application in SCD management [52].

Novel Therapeutics in Preclinical Development

Emerging preclinical strategies for SCD extend beyond canonical ex vivo gene therapies and encompass a diverse array of molecular, pharmacologic, and in vivo genomic approaches aimed at modulating hemoglobin expression, mitigating red cell pathology, and attenuating vascular complications.

Epigenetic Modulation of HbF

Targeted epigenetic remodeling has become a focal point for reactivating γ-globin gene expression and restoring HbF synthesis. Small-molecule inhibitors directed against chromatin regulators, such as lysine-specific demethylase 1 (LSD1), histone deacetylases (HDACs), DNA methyltransferases (DNMTs), and protein arginine methyltransferases (PRMTs), have demonstrated efficacy in derepressing HbF loci in preclinical erythroid systems. These compounds function by disrupting repressive histone and DNA methylation patterns, thereby enhancing transcriptional accessibility of the HBG genes. Parallel strategies targeting key transcriptional repressors of HbF, including ZBTB7A, SOX6, KLF1, and ZNF410, are under investigation to further fine-tune HbF induction at the level of gene regulation [17,22].

Metabolic and Oxygen Affinity Modifiers

Metabolic modulation of red cell physiology is another promising therapeutic axis. Pyruvate kinase activators augment glycolytic flux, increasing red cell ATP and 2,3-DPG levels to improve membrane integrity and reduce intracellular sickling under hypoxic stress. Allosteric hemoglobin modifiers, by stabilizing the oxygenated hemoglobin conformation, inhibit the polymerization of deoxygenated sickle hemoglobin, preserving erythrocyte deformability and prolonging circulatory lifespan [18,44].

Anti-adhesion and Anti-inflammatory Strategies

The adhesive interactions between erythrocytes, leukocytes, and endothelium are critical mediators of vaso-occlusion. Novel biologics, including humanized monoclonal antibodies targeting P-selectin and other adhesion molecules, aim to disrupt these pathologic cell-cell interactions. In parallel, anti-inflammatory compounds such as adenosine A2A receptor agonists and omega-3 fatty acids are being explored to attenuate endothelial activation, neutrophil priming, and inflammatory cytokine release. Complementary anticoagulant approaches, including low-molecular-weight heparins like tinzaparin, seek to address the prothrombotic state that exacerbates vaso-occlusion and tissue ischemia in SCD [18,44,51,83-85].

In Vivo Gene Therapy Platforms

A transformative frontier in the field involves the direct in vivo delivery of gene editing or gene modulation constructs, obviating the need for myeloablation and ex vivo cell processing. Viral and non-viral delivery platforms, such as lipid nanoparticles and AAV vectors, are being optimized to selectively transduce hematopoietic stem and progenitor cells in situ. These technologies aim to simplify logistics, reduce procedural morbidity, and expand the accessibility of curative therapies to broader global populations with limited infrastructure [51,83-87].

Together, these preclinical innovations represent a multifaceted and mechanistically diverse pipeline poised to reshape the therapeutic paradigm for SCD through targeted, durable, and scalable interventions.

Improving Accessibility and Affordability

The scalability and equitable dissemination of gene therapies for SCD are contingent upon transformative shifts in manufacturing infrastructure, economic frameworks, and global policy coordination.

Manufacturing Innovation and In Vivo Platforms

A primary driver of cost reduction lies in the development of streamlined and scalable manufacturing platforms. Transitioning from complex ex vivo protocols to in vivo gene editing strategies, utilizing targeted delivery of gene editing machinery directly to hematopoietic stem cells, has the potential to eliminate the need for autologous cell collection, conditioning regimens, and centralized manufacturing. This paradigm shift may dramatically simplify logistics, reduce procedural morbidity, and lower per-treatment costs while maintaining therapeutic efficacy [49]. 

Alternative Payment Models and Financing Structures

In parallel, novel reimbursement strategies are under active exploration to mitigate the upfront economic burden of gene therapies. These include annuity-based payment models that distribute cost over time and outcomes-based agreements that tie payment to long-term clinical benefit. Such models aim to align incentives across payers, providers, and manufacturers, particularly in publicly funded systems where short-term budget impacts may hinder adoption [83,84].

Global Access and Policy Harmonization

Addressing the global inequity in SCD treatment requires coordinated policy efforts and public-private partnerships. International harmonization of regulatory standards, expansion of technology transfer programs, and investment in regional manufacturing hubs are critical to enabling access in low- and middle-income countries where the burden is highest. Frameworks promoting equitable intellectual property licensing, capacity-building initiatives, and collaborative research consortia are essential to foster global inclusivity in gene therapy deployment [38].

These accessibility-driven strategies complement ongoing therapeutic innovations, forming the foundation for a sustainable, globally relevant approach to the long-term management and potential cure of SCD.

## Conclusions

Gene therapy for SCD has advanced rapidly, with lentiviral vectors enabling gene addition and CRISPR/Cas9 approaches reactivating fetal hemoglobin or repairing the β-globin gene. Clinical trials demonstrate encouraging outcomes, including transfusion independence, durable hemoglobin increases, and significant reductions in vaso-occlusive crises, bringing the prospect of a functional cure closer to reality. Emerging in vivo strategies may further streamline therapy by bypassing ex vivo stem cell manipulation, offering a simpler and potentially more scalable approach.

Despite these breakthroughs, major challenges persist. The high cost of treatment, often exceeding $1.5 million, along with the need for intensive conditioning regimens and specialized infrastructure, limits accessibility, particularly in LMICs where the burden of SCD is greatest. Achieving equitable access will require global efforts to reduce costs, expand healthcare capacity, and establish ethical and regulatory frameworks to ensure that these transformative therapies reach all patients in need.
